# Criticality and Magnetic Phases of Ising Shastry–Sutherland Candidate Holmium Tetraboride

**DOI:** 10.3390/ma18112504

**Published:** 2025-05-26

**Authors:** Guga Khundzakishvili, Bishnu Prasad Belbase, Pravin Mahendran, Kevin Zhang, Hanjing Xu, Eliana Stoyanoff, Joseph George Checkelsky, Yaohua Liu, Linda Ye, Arnab Banerjee

**Affiliations:** 1 Department of Physics and Astronomy, Purdue University, West Lafayette, IN 47907, USA; 2 Department of Computer Science, Purdue University, West Lafayette, IN 47907, USA; 3 Department of Physics, Massachusetts Institute of Technology, Cambridge, MA 02139, USA; 4 Neutron Scattering Division, Oak Ridge National Laboratory, Oak Ridge, TN 37831, USA; 5 Department of Physics, Stanford University, Stanford, CA 94305, USA; 6 Division of Physics, Mathematics and Astronomy, California Institute of Technology, Pasadena, CA 91125, USA

**Keywords:** neutron scattering, magnetization, susceptibility, Frustration, shastry-sutherland lattice, simulated annealing

## Abstract

Frustrated magnetic systems arising in geometrically constrained lattices represent rich platforms for exploring unconventional phases of matter, including fractional magnetization plateaus, incommensurate orders and complex domain dynamics. However, determining the microscopic spin configurations that stabilize such phases is a key challenge, especially when in-plane and out-of-plane spin components coexist and compete. Here, we combine neutron scattering and magnetic susceptibility experiments with simulations to investigate the emergence of field-induced fractional plateaus and the related criticality in a frustrated magnet holmium tetraboride (HoB_4_) that represents the family of rare earth tetraborides that crystalize in a Shastry–Sutherland lattice in the ab plane. We focus on the interplay between classical and quantum criticality near phase boundaries, as well as the role of material defects in the stabilization of the ordered phases. We find that simulations using classical annealing can explain certain observed features in the experimental Laue diffraction and the origin of multiple magnetization plateaus. Our results show that defects and out-of-plane interactions play an important role and can guide the route towards resolving microscopic spin textures in highly frustrated magnets.

## 1. Introduction

Frustrated magnetic materials exhibit some of the most exotic and poorly understood phases in condensed matter physics, driven by competing interactions that prevent conventional magnetic order [1,2,3]. Lattices such as the Shastry–Sutherland lattice (SSL), Kagome and triangular lattices host a variety of unconventional phases, including fractional magnetization plateaus and incommensurate orders [4,5,6]. Understanding the microscopic spin configurations responsible for these emergent phases, and their evolution with external tuning parameters such as magnetic field and temperature, remains a central challenge in frustrated magnetism [7]. These questions are not only of fundamental interest but are also directly relevant to modern efforts in designing functional quantum material-based platforms for emergent phenomena like topological order and quantum spin liquids [8,9].

Among these systems, the rare earth-based SSL magnets HoB_4_, TmB_4_ and ErB_4_ provide an attractive experimental platform, with their strong single-ion anisotropy combined with large magnetic moments [4,10,11]. These systems exhibit a series of fractional magnetization plateaus, such as $\frac{M}{M_{sat}}=\frac{1}{2},\;\frac{1}{3},\;\frac{1}{7},\frac{1}{9}$ under an applied field, reflecting non-trivial spin textures stabilized by geometric frustration [12,13]. However, early studies often interpreted these in terms of simple collinear out-of-plane spin configurations [14]. Yet growing experimental evidence, such as the studies of neutron diffraction and magnetometry, suggests a much richer picture involving in-plane spin components, domain formation and complex stripe-like orders [15,16].

Additionally, many frustrated systems exhibit signatures of criticality—both classical critical fluctuations near finite-temperature phase boundaries and quantum criticality near field-induced transitions at low temperatures [17,18]. Disentangling the role of classical vs. quantum critical phenomena in these frustrated lattices and understanding how they connect to the formation of fractional plateaus, and the role of domain dynamics, remains an open question [19,20]. It is important to understand whether a new phase appears from quantum origins or from sub-leading terms in the Hamiltonian or perhaps, defects. Such insights are crucial for understanding how magnetically frustrated systems respond to external fields and how they can be tuned toward novel quantum phases.

In this work, we combine field-dependent neutron scattering and simulated annealing to study the emergence of fractional plateaus and critical phases in a single crystal of the frustrated Shastry–Sutherland candidate HoB_4_. We explore the field-temperature phase diagram, focusing on the interplay between in-plane and out-of-plane order, domain dynamics and new features that emerge near phase transitions. We discover a new phase—the only one with in-plane magnetic Bragg reflections in HoB_4_—close to the critical (C) phase boundary of the ordered antiferromagnetic (AFM) and high temperature paramagnetic phases, which are unreported in the literature. From the computationally derived spin patterns, we reveal that in-plane order is intricately affected by inter-layer interactions, especially close to a phase transition, which are qualitatively similar to the features observed in HoB_4_ neutron scattering data. Our results show the qualitative effects of the non-ideal terms of the Hamiltonian—the out-of-plane magnetic superexchange interactions and the defects—on the in-plane order in a frustrated spin system. This path presents a unique approach for resolving microscopic order in complex magnetic systems, with implications for a broad class of frustrated quantum magnets. Our results point to complex ordered phases and a delicate phase diagram affected by a variety of terms in the Hamiltonian close to the phase transition, which are difficult to explore just using analytical techniques. We propose this system as an excellent candidate for developing Hamiltonian discovery kernels driven by machine learning (ML) platforms for Hamiltonian discovery and inverse scattering problems [21,22].

## 2. Shastry–Sutherland Model, HoB_4_ Crystal Synthesis and Structure

**Figure 1:**
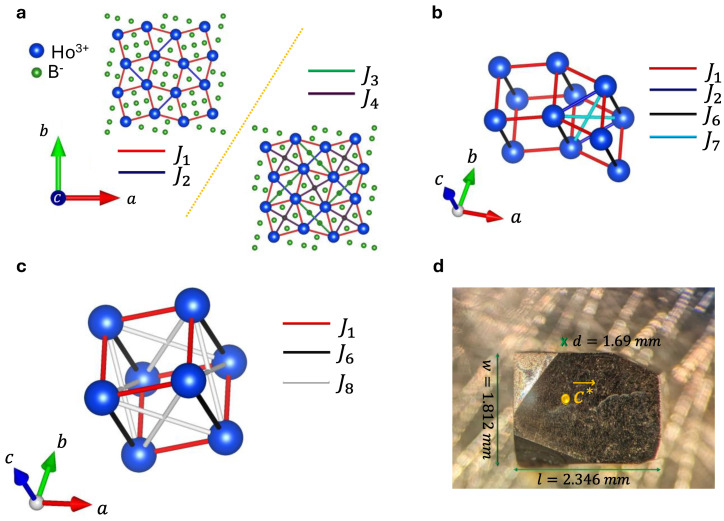
Lattice of Holmium atoms in ${\mathrm{HoB}}_{4}$ single crystal. (**a**) In-plane structure of $HoB_{4}$ single crystal. Top corner corresponds to in-plane lattice with only $J_{1}$ and $J_{2}$ interactions topologically equivalent to SSL lattice. Bottom corner displays additional in-plane $J_{3}$ and $J_{4}$ interactions. $J_{5}$ interaction is not shown here but depicted in Figure S1. (**b**,**c**) Out-of-plane $J_{6},\;J_{7},\;J_{8}$ interactions. (**d**) Single-crystal $HoB_{4}$ with mass of m=61.2 mg.

The Shastry–Sutherland model is a paradigmatic model in quantum magnetism because of its exact solvability. In this model, the spins are arranged on the square lattice with additional diagonal bonds on alternate plaquettes (a HoB_4_ based topologically equivalent lattice, with red and blue lines for $J_{1}$ and $J_{2}$ bonds correspondingly in Figure 1a), which can be expressed with the following Hamiltonian:

(1) $${\hat{H}}_{SSL}=J_{1}\underset{i,j}{\sum }S_{i}S_{j}+J_{2}\underset{k,l}{\sum }S_{k}S_{l}$$

The first summation corresponds to the nearest neighbors, while the second one corresponds to diagonal bonds on alternate plaquettes. This additional arrangement of the alternate plaquettes introduces geometric frustration. This exact solution provides profound insights into the interplay between frustration and quantum fluctuations in low-dimensional quantum systems [23]. The Heisenberg variant of the SSL model encompasses comprehensive quantum spin interactions and accommodates intricate states like dimer–singlet ground states, magnetization plateaus, thermal Hall plateaus and even quantum spin liquids under specific conditions [24,25,26], and it is studied extensively experimentally for the search of exotic plaquette phases and magnetization plateaus, especially in SrCu_2_(BO_3_)_2_ [27,28,29,30]. The Ising cousin of this model is easier to compute given the commuting of Ising spins, which makes it a simpler first system to understand intricate details of out-of-plane interactions and defects. Examples of the Ising model include TmB_4_, NdB_4_ and HoB_4_. Conversely, the Ising model—where spins are confined to distinct orientations—provides a computationally manageable approach to comprehending the effects of frustration, defects and notably out-of-plane interactions, which pose challenges in the complete quantum framework. Compounds such as TmB_4_ and NdB_4_ effectively realize this Ising limit, exhibiting magnetization plateaus and commensurate spin structures. In this regard, HoB_4_ emerges as a candidate system: it exhibits intermediate behavior, merging robust Ising anisotropy with supplementary degrees of freedom from its substantial angular momentum (In the absence of spin–orbit coupling (SOC) and crystal electric field (CEF) splitting J=8 for ${\mathrm{Ho}}^{3+}$), positioning it as a promising platform for connecting the Ising and Heisenberg interpretations of the SSL and for examining field-tuned classical and quantum criticality.

Single crystals of HoB_4_ have been synthesized using the floating zone method. Polycrystalline Ho^11^B_4_ was first synthesized by reacting thoroughly mixed and compressed Ho_2_O_3_ and ^11^B_4_ powder in an argon gas flow. The resulting polycrystalline rods were then zone-refined to obtain high-quality single crystals. To determine the crystal structure, single-crystal X-ray diffraction (XRD) data were collected in a single-crystal X-ray laboratory in the chemistry department of Purdue University using a Bruker D8 Quest diffractometer equipped with Mo Kα radiation (λ=0.71073 Å). Measurements were performed at room temperature. The results of the single-crystal XRD are presented in Table 1. The crystal magnetic lattice is shown in Figure 1a–c and conforms to an SSL with lattice constants a=b=7.01 Å and c=4.01 Å. The consequent reciprocal lattice vectors $a^{*}$, $b^{*}$, $c^{*}$ are $a^{*}=b^{*}=\frac{2\pi }{a}=0.88\;{Å}^{-1}$ and $c^{*}=\frac{2\pi }{c}=1.57\;{Å}^{-1}$, respectively.

In Figure 1a–c, the SSL interactions are denoted by $J_{1}$ and $J_{2}$*,* providing the primary mechanism for frustration, which makes the model topologically equivalent to the Shastry–Sutherland model. Additional interactions, which deviate from the simple Shastry–Sutherland type, are also expected to be present in a realistic situation, and which are also represented in Figure 1a–c, such as additional in-plane interactions $J_{3}$*–*$J_{5}$ and out-of-plane ones $J_{6}$–$J_{8}$, and these are discussed more in Section 5.

A small number of defects and stacking faults can change the exact nature of the phases, especially close to criticality, as well as the phase diagram in 2D crystals. The exact nature of the phase diagram is often found to be sample-dependent, and it is difficult to reconcile measurements performed using different techniques on different samples—especially for the delicate phases. Additionally, for large samples with a large effective magnetic moment, the demagnetization factors are different for different sample shapes, making it hard to reconcile measurement and phase diagrams from different measurements. Unlike previous measurements on HoB_4_, for this manuscript, we have performed all the measurements, both neutron diffraction and susceptibility/magnetization measurements on the same single crystal of HoB_4_ (Figure 1d), allowing us a direct comparison of the results.

## 3. Magnetic Susceptibility Measurements

Magnetization/susceptibility measurements are a staple technique to divulge magnetic phases and magnetic phase transitions. To deduce the phase diagram and critical regimes in HoB_4_, we performed magnetic susceptibility measurements using the Superconducting Quantum Interference Device (SQUID) magnetometer (MPMS-3 Quantum Design, San Diego, CA, USA), equipped with an ^4^He insert at the BIRCK Nanotechnology Center in Purdue University with a base temperature of T=1.8 K. The measurement was conducted in two separate parts: (1) Magnetic field sweep for fixed temperature and applied magnetic field along c ($c^{*}$) axis, yielding M vs. B dependence; the magnetization value is normalized as $M\to M/M_{sat}$, where $M_{sat}$ represents saturated magnetization (in the units of $\frac{\mu_{B}}{{\mathrm{Ho}}^{3+}}$) for an applied saturation magnetic field. (2) Temperature sweep for the fixed applied magnetic field along the c ($c^{*}$) axis, extracting magnetic susceptibility $\chi =\frac{M}{H}$ vs. T.

**Figure 2:**
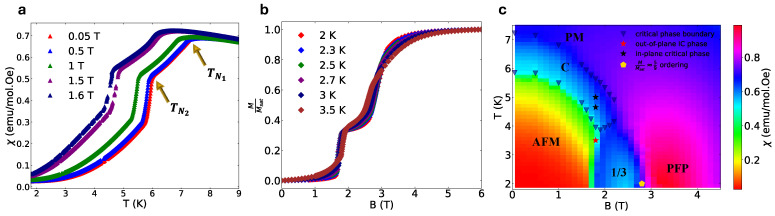
(**a**) Magnetic susceptibility as a function of temperature, displaying two consecutive phase transitions between paramagnetic (PM) and low-temperature antiferromagnetic (AFM) phases. Figure S2 displays a detailed phase transition analysis for the applied B=0.05 T magnetic field. (**b**) Magnetization normalized with saturation magnetization as a function of the applied field, displaying a 13 magnetization between AFM on low fields and partially field-polarized (PFP) phases on high fields. The 2 K magnetization data on Figure S3 depict the trace of additional magnetization ($M/M_{sat}\approx 5/9$) that appears between the 13 and PFP phases. (**c**). Magnetic phase diagram of $HoB_{4}$ reconstructed from magnetic susceptibility measurement, displaying following phases: AFM, PM, PFP, $M/M_{sat}=1/3$, $M/M_{sat}\approx 5/9\;$ and an in-plane classical critical phase (C) that supports the in-plane order (details in text).

The sensitive nature of the phase diagram in HoB_4_ is immediately apparent in its magnetic susceptibility, which provides us with additional information regarding phase transitions. The analysis of phase transitions is split into several different regions. As shown in Figure 2a, by the evolution of magnetic susceptibility at the zero field, the sample undergoes two discontinuities at $T_{N_{1}}=7.22\;K$ and $T_{N_{2}}=5.97\;K$. The $T_{N_{1}}$ and $T_{N_{2}}$ vary with the application of the out-of-plane magnetic field, clearly defining the boundary of the low-temperature phase. The low-temperature phase shows signatures of an AFM spin arrangement, as inferred from the decreased susceptibility of the sample to the applied magnetic field. The phase transition at $T_{N_{1}}$ (between the high-temperature paramagnetic and intermediate critical phase) displays a broader maximum pointing towards the second order in nature. Conversely, for the phase transition at $T_{N_{2}}$, magnetic susceptibility experiences a sharp drop, which suggests a likely first-order phase transition to the AFM phase.

The results are summarized in Figure 2 on the phase diagram constructed from the combination of all the magnetization measurements. It reveals the paradigmatic features of a quantum phase transition with the following phases: (1) an AFM phase at low fields, (2) a ‘partially field-polarized’ (PFP) phase at high fields and (3) the one-third magnetization phase emerging close to the critical point of the AFM→PFP phase transition. The triangles, which represent phase transition temperatures and fields derived from susceptibility analysis, outline the boundary of the classical critical (C) phase sandwiched between $T_{N_{1}}$ and $T_{N_{2}}$ that appears above the phase boundary of the AFM phase. The one-third magnetization plateau forms around B =1.7 T and sustains up until B =2.8 T (Figure 2b). As we discuss in the next section, we find this phase coexists with an incommensurate spin-ordered phase that sustains to even higher temperatures and fields, and the phase marked PFP supports only the incommensurate order that exists everywhere except for in the AFM phase.

The phase diagram observed by us in the susceptibility measurements is simpler than the one proposed earlier. Besides the one-third plateau in the vicinity of the AFM→PFP transition, earlier sources [31,32] had claimed that additional magnetization plateaus can be observed in the narrow ranges of the magnetic field for the temperatures at and below 2 K, such as $\frac{M}{M_{sat}}=\frac{1}{2};\frac{4}{9};\frac{3}{5}$ magnetization plateaus. Our magnetization measurements conducted on single-crystal HoB_4_ instead just reveals a trace of a plateau at roughly $\frac{M}{M_{sat}}=0.55\;≅\frac{5}{9}\;$ between the one-third plateau and PFP phase (Figure S3), which is marked with a golden pentagon in Figure 2c. Overall, comparing our data with [31,32,33,34], we can also conclude that some of the plateaus could be non-universal and sample-dependent. Whether these are stabilized in defects and different out-of-plane interactions or quantum fluctuations are possibilities we will address more in Section 5.

## 4. Neutron Diffraction

Neutron Laue diffraction provides the static structure factor, which is a robust tool for determining the phases of magnetic materials, revealing magnetization plateaus and commensurate/incommensurate magnetic orders. It is a robust tool for understanding the delicate phases that arise at critical regimes of a phase diagram. Neutron diffraction measurements on a single-crystal HoB_4_ (Figure 1d) were performed at Spallation Neutron Source (SNS), Oak Ridge National Laboratory (ORNL), at the Elastic Diffuse Scattering Spectrometer CORELLI, with an incident neutron energy range of $E_{i}=10-200\;\mathrm{meV}$. CORELLI employs a white-beam Laue diffraction method, allowing for the simultaneous collection of diffraction data across a broad range of wavelengths. To discriminate between elastic and inelastic scattering, CORELLI utilizes a statistical chopper that modulates the incoming neutron beam quasi-randomly. The resulting data are processed using a cross-correlation method, which reconstructs the elastic scattering signal by correlating the modulated incident beam with the detected scattered neutrons. This approach effectively isolates elastic components from the total scattering data. The sample was aligned in the ab (i.e., in the 2D Shastry–Sutherland) plane with the magnetic field pointing parallel to the c ($c^{*})$ axis and rotated in the range of ±60° around the c axis, with a step size of 2°.

**Figure 3:**
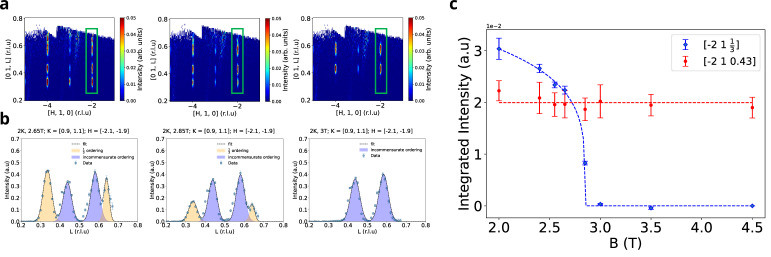
Neutron diffraction data in $\left(H\;1\;L\right)$ planes. (**a**) Evolution of 1/3 and incommensurate orders in range of applied field 2.65–3 T; 2D slices have been made for $K=\left[0.9,\;1.1\right]$ r.l.u integration range. (**b**) Evolution of 1/3 and incommensurate orderings in range of applied field 2.65–3 T; 1D slices have been made for $K=\left[0.9,\;1.1\right]\;r.l.u$ and $H=\left[0.9,\;1.1\right]\;r.l.u$ integration range. (**c**). Integrated peak intensities as function of applied magnetic field.

We first concentrate on the location of the one-third magnetization plateau in the field regime B=2.65−3.0 T at T=2 K. Figure 3a shows the data on the [H K=1 L] slice, where K is integrated between [0.9, 1.1] r.l.u. We immediately notice that the magnetic Bragg peaks remain at integer locations along [H, K], i.e., on the ab plane. However, in the out-of-plane or the stacking direction, we reveal peaks at fractional indices: the ferrimagnetic phase $L=\left(\frac{1}{3}\pm 3\cdot 10^{-4}\right)\;$ r.l.u and incommensurate phase with $L_{inc}=(0.43\;\pm 0.012$) r.l.u in Figure 3a. The results are clarified further in Figure 3b, where we provide a cut along [−2 1 L] that is fitted to two Q-vectors at L=0.43 r.l.u and L=0.33 r.l.u (and their Fourier conjugate peaks at L=0.57 r.l.u and L=0.67 r.l.u). We observe no additional magnetic Bragg peaks in the c direction in neutron scattering. Most of the features are consistent with the one-third and the 0.43 orders that were reported earlier [31]. We performed a meticulous search for the additional phases, such as narrow magnetization plateaus at $\frac{M}{M_{sat}}=\frac{1}{2};\frac{4}{9};\frac{3}{5}$ reported earlier, and found a trace of none. However, a trace of $\frac{M}{M_{sat}}\approx \frac{5}{9}$ ordering has been established at T=2 K (Figure S3). Thus, it is reasonable to conjecture that the existence of certain magnetization plateaus in HoB_4_ can be specific to the sample and could arise in the presence of non-universal defects, as is discussed in Section 5 of this study.

Figure 3c depicts the evolution of intensities of magnetic Bragg peaks related to out-of-plane magnetic orders. The scattering intensities for the incommensurate Bragg peak [−2 1 0.43] sustains to large magnetic fields, while ferrimagnetic commensurate ordering [−2 1 1/3] diminishes as a function of the applied magnetic field and fully disappears in the vicinity of B=3 T.

Notably, the integrated intensity of the incommensurate phase is unaffected through the diminishing of the one-third order leading into the partially field-polarized phase at 3 T (Figure 2c). This is particularly surprising, given that HoB_4_ has only one Holmium site, with the overall ordered moment conserved, and hence it is natural to expect a competition between long-range orders and a trade-off between net ordered moments of different Q-vectors. Our results instead show that the incommensurate phase is robust and derives from mechanisms (or terms in the Hamiltonian) distinct from the one-third, C and PFP phases.

In the next section, we concentrate on the regime of classical criticality intermediate between $T_{N_{1}}$ and $T_{N_{2}}$ in the susceptibility phase diagram in Figure 2c. Our motivation was driven by a goal to discover new phases close to the critical regime, where the enhanced effects of long-range fluctuations can lead to the genesis of a new in-plane order where sub-leading terms of the Hamiltonian play a more defining role. In-plane indexed peaks have been observed in other rare-earth SSL candidates such as NdB_4_ [13] and TmB_4_ [14]. The critical phase turns out to be good place to find such phases in HoB_4_, because strong fluctuations could enhance the delicate sub-leading terms of the Hamiltonian.

**Figure 4:**
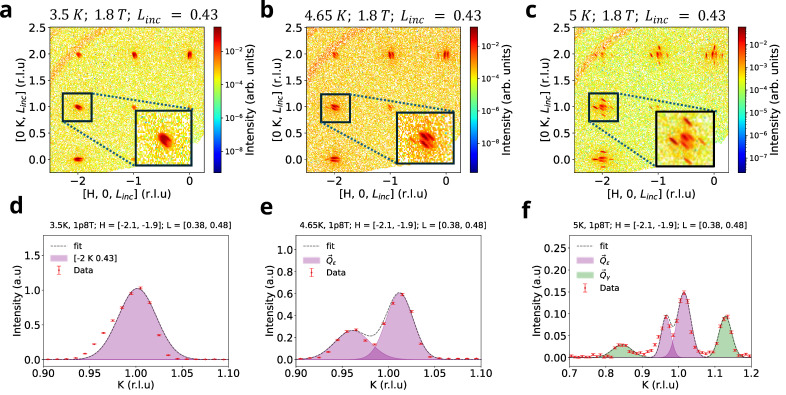
In-plane ordering of critical intermediate phase. 2D slice of $\left(H\;K\;L_{inc}\right)$ has been made with $L_{inc}=\left(0.43\;\pm 0.05\right)$ r.l.u integration range at 1.8 T field and [3.5, 4.65, 5] K temperatures. (**a**–**c**) Emergence of ($0,\;\pm \varepsilon ,\delta^{\prime }$) and $\left(0,\pm \gamma ,\delta^{\prime }\right)$ ordering of incommensurate peak splitting as the function of temperature. (**d**–**f**) A 1D cut on the K reciprocal direction for $H=\left(-2\;\pm 0.1\right)$ r.l.u integration range. ($0,\;\pm \varepsilon ,\;\delta^{\prime }$) ordering with $\varepsilon =\left(0.0245\;\pm \;0.004\right)$ r.l.u $\delta^{\prime }=0.43$ r.l.u and $\left(0,\pm \gamma ,\delta^{\prime }\right)$ with γ = 0.14 r.l.u. $\frac{1}{7}$ ordering exists in the vicinity of 5 K, 1.8 T and is absent for 4.65 K, 1.8 T. Incommensurate out-of-plane ordering sustains throughout the entirety of the intermediate phase between $T_{N_{1}}$ and $T_{N_{2}}$.

At the critical regime (black stars in Figure 2c), we discover a new phase corresponding to a magnetic Bragg peak splitting into in-plane commensurate and incommensurate orderings. The transition into the critical phase is further explained in the slices of $\left(H\;K\;0\right)⊥c^{*}$ reciprocal planes for B=1.8 T. As has been shown in in-plane neutron diffraction measurements in Figure 4a–c, upon increasing the temperature from 3.5 K to 5 K, we report two separate in-plane orders: the evolution of in-plane magnetic order that results in $\left[H\;K\;L_{inc}\right]$ splitting into ${\vec{Q}}_{\varepsilon }=\left(\pm \varepsilon ,0,\delta^{\prime }\right)$ and ${\vec{Q}}_{\varepsilon }=\left(0,\pm \varepsilon ,\delta^{\prime }\right)$ satellite peaks along the *H* and *K* directions correspondingly (Figure 4b), where ε=(0.0245 ± 0.004) r.l.u and $\delta^{\prime }=0.43$ r.l.u. This is accompanied by another new set of satellite peaks with the modulation vectors ${\vec{Q}}_{\gamma }$ = $\left(0,\pm \gamma ,\delta^{\prime }\right)$ and ${\vec{Q}}_{\gamma }$ = $\;\left(\pm \gamma ,0,\delta^{\prime }\right)$ at T=5 K (Figure 4c). We fit the cut along K at the [−2 K 0.43] in Figure 4f, leading to $\gamma =0.142\pm 0.00025\approx \frac{1}{7}$ r.l.u. Thus, at the critical point we report on the discovery of a, likely commensurate, in-plane H=K=1/7 ordering. The corresponding modulation vector for the order is $\vec{Q}={\vec{Q}}_{\varepsilon }\;⊕\;{\vec{Q}}_{\gamma }$. We note that this is the only in-plane order in HoB_4_, appearing as an additional modulation of the incommensurate order along $c^{*}$.

The signature of field-induced $\left(\pm \gamma ,\pm \gamma ,\;\delta^{\prime }\right)$ ordering with in-plane indices exists in a very narrow range or temperature and exists in the critical phase shown in Section 3. If such a splitting of in-plane ordering can be triggered by out-of-plane interactions between $Ho^{3+}$ ions, which consists of superexchange and dipole–dipole interactions, is qualitatively discussed in the next section.

Overall, the results show that the $L_{inc}=\delta^{\prime }=0.43$ r.l.u phase is absent only in the AFM phase but otherwise particularly robust in HoB_4_. This robustness suggests that it arises from leading terms of the effective Hamiltonian. The other orders, such as the one-third plateau, are sub-leading effects. The AFM phase trades intensity into the incommensurate order. It is therefore important to deduce the nature of the incommensurate phase, using a 3D model where frustration is not only from in-plane interactions but also arises in strong out-of-plane interactions, before an analysis of the other sub-leading orders codependent on the incommensurate phase would be possible. While the exact origin of the $L_{inc}=0.43$ incommensurate order is still a mystery, a qualitative attempt to understand the origins of several other features of the critical phase is presented next.

## 5. SSL Simulation

The 2D Ising SSL model is shown in Equation (1). This 2D SSL model has been analyzed in detail, first by Dublenych [35] with no additional interactions and then in [36] in the presence of additional interactions, where new plateaus $\frac{M}{M_{sat}}=\frac{1}{9},\frac{1}{6},\frac{2}{3},\frac{4}{9},\frac{1}{2}\;$ with an in-plane order were theoretically observed. Additionally, this model represents an appealing platform for studying long-range ordering and continuous and discontinuous quantum phase transitions in 2D frustrated magnets [37,38,39]. The results are also analyzed and reproduced using D-Wave quantum annealer [40], where the classical ground state and the phase transitions were reproduced and spin behavior at the critical points predicted. Later, in [41] the expanded version of the SSL Hamiltonian (the first and third summation of Equation (2)) was analyzed using a Fujitsu parallel tempering machine to discover a host of new phases, such as the in-plane 5/9th magnetization plateau, that does not exist in the just a $J_{1}-J_{2}$ model. We note that the prior analysis is limited to the 2D Ising SSL which is insufficient for HoB_4_, since many of the ordered phases observed by us are out-of-plane. The in-plane order that we observed is in the envelope of the out-of-plane incommensurate phase $L_{inc}=0.43$ r.l.u., also underscoring the importance of a full 3D treatment.

We also note here that HoB_4_ has low resistance at low temperatures and behaves like a metal [42]. The conducting electrons would hybridize the spins and can lead to long-range interactions, which are commonly known as RKKY interactions. The role of RKKY interactions for those Ising Shastry–Sutherland candidates has been discussed earlier in references [36,43]. It was found there that some of the long-range ordered phases, such as $M=\frac{1}{2}$, may originate not only from higher-order interaction terms beyond the Shastry–Sutherland but could also arise as a result of the RKKY interactions. Whether RKKY coupling between the localized 4F electrons and 6S conduction electrons could be a dominant mechanism in HoB_4_, and whether this mechanism can explain some of the incommensurate orders observed, remains an open question and an interesting area for future study.

Questions arise whether (a) the features observed in HoB_4_ can be described by an Ising Hamiltonian, and (b) whether an SSL Hamiltonian with out-of-plane interactions may be able to capture some of the observed features. To answer the first question, we refer to Figures 7 and 9 in ref. [31] and the discussion therein. The AFM phase has a spin canting 23° away from the *c* axis; thus, our field along the *c* axis has a sizeable transverse component making perhaps, a transverse-field Ising model more appropriate. On the other hand, the phases that support the incommensurate, C, one-third and PFP phases all have spin moments along the c axis. Given that our field B$\;||\;c^{*}$ is hence a longitudinal field, these phases observed in HoB_4_ could be compliant to an Ising SSL model with a longitudinal field.

To answer the second question, while a full treatment of the incommensurate out-of-plane order will require a very large 3D lattice, which could be very useful future work, we show that a longitudinal-field Ising 3D SSL model (i.e., a 2D SSL model extended with out-of-plane interaction terms), inspired by the XRD data of HoB_4_ single crystal, can qualitatively capture some of the features we observed in the neutron scattering data in the critical phase.

We consider an extended SSL model, starting with the results of [41] but with additional out-of-plane interaction terms to seek the behavior of the spin–spin correlations close to a phase transition:

(2) $$\hat{H}=\overset{5}{\underset{i=1}{\sum }}J_{i}\underset{k,\;l}{\sum }S_{k}S_{l}+\overset{8}{\underset{i=6}{\sum }}J_{i}\underset{k,\;l}{\sum }S_{k}S_{l}-h\underset{m}{\sum }S_{m}$$

The first term including $J_{1}–J_{5}$ represents the in-plane interactions depicted in Figure 1a. The second term including $J_{6}–J_{8}$ is the out-of-plane interactions shown in Figure 1b,c. The out-of-plane interaction $J_{6}$ is parallel to the c axis and represents a direct out-of-plane connection between ab planes. The $J_{7}$ and $J_{8}$ interactions are located under $J_{2}$ and $J_{1}$, respectively. We additionally implement a Zeeman coupling of the out-of-plane magnetic field with individual spin sites denoted by the last term of Equation (2) (m runs over the entire spin array). The corresponding magnetic phases can be identified by mapping $M=\langle S_{m}\rangle$ in $\left(J_{i},\;h\right)$ nine-dimensional parameter space, which afterwards is translated into M vs. h for appropriate $J_{i}$ values.

Starting with spin–spin interaction values ($J_{i}$) inspired from experimental data, we perform a simulated annealing and parallel tempering on the Neal simulated annealer offered by D-Wave, which allows us to explore an Ising Hamiltonian with arbitrary connectivity. We compute the ground states of Equation (2) and compare their magnetization values with the applied transverse magnetic field, following the same steps in [35,36]. The existence of magnetization plateau(s) can be confirmed by plotting magnetization as the function of the applied magnetic field for fixed $J_{i}$ values. The direction of the ordering can be determined by a fast Fourier transform (FFT) of the extracted spin structure from the lattice spin state. Fourier analysis of spin structure yields information about the symmetry of the system of spin arrangements, which qualitatively carries the same information as magnetic Bragg peaks in neutron diffraction measurements. Once the magnetization plateau is determined, we extract the in-plane and out-of-plane spin structure factors via FFT of the two-spin correlation function.

Due to the large magnetic moment of Ho^3+^, dipole–dipole interaction is an important part of the overall interactions between spin sites. We extract the initial parameters of the magnetic Hamiltonian in Equation (2) from the interionic distances of Ho^3+^ (Table 2) using the inverse cubic nature of magnetic dipole–dipole interaction:

(3) Ud−d~S1S2 r3

The initial target values for spin–spin interactions (which themselves include both dipole–dipole and exchange interactions) thus inferred are presented in Table 2 from XRD measurements.

**Figure 5:**
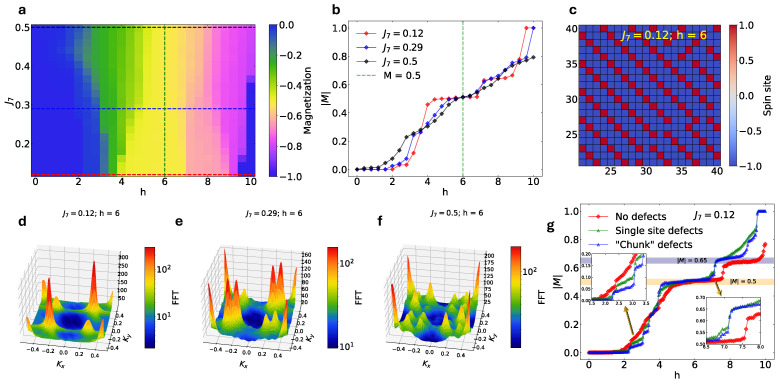
(**a**) Phase diagram for variable $J_{7}$ and h in the absence of defects. The rest of the parameter values: $J_{1}=1,\;J_{2}=1.03,\;\;J_{3}=0.19,\;\;J_{4}=0.35,\;J_{5}=0.14,\;J_{6}=0.77,\;J_{8}=0.308$. (**b**) The evolution of magnetization plateau $\left|M\right|=0.5$, as the function of $J_{7}$ in the absence of defects. (**c**) In-plane $\left|M\right|=0.5$ magnetic ordering for $J_{1}=1,\;\;J_{2}=1.03,\;\;J_{3}=0.19,\;J_{4}=0.35,\;\;J_{5}=0.14,\;\;J_{6}=0.77,\;\;J_{7}=0.12,\;\;J_{8}=0.308,\;h=6$, showing spin down–down–down–up ordering for the middle part of layer 5 (the system consists of 60×60×9 spins). (**d**–**f**) Fourier transform of spin structures for $J_{7}=\left[0.12,\;0.29,\;0.5\right]$ displaying the evolution of in-plane magnetic ordering as the function of out-of-plane interaction $J_{7}$ in the absence of defects. (**g**) Magnetization evolution for $J_{7}=0.12$ with and without defects, displaying the appearance of additional magnetization plateaus when single and ‘chunk’ defects are introduced.

We implement five in-plane and three out-of-plane spin–spin interactions (Figure 1a–c) using a lattice size of 60×60×9=5400 spins. Spin–spin interaction parameters are initialized based on Table 2 and varied within a ±60% range. For visualization purposes, we plot separate phase diagrams as colormaps on $J_{i}$ vs. h for $i\epsilon \;\left[2,\;8\right]$ ($J_{1}=1$). Analysis of phase diagram(s) reveals a magnetization plateau at $\left|M\right|=0.5$ (Figure 5a, marked with a yellow color). In Figure 5b, it is evident that the one-half magnetization plateau becomes less pronounced as we increase the out-of-plane interaction parameter $J_{7}$. The corresponding spin arrangement down–down–down–up (Figure 5c) evolves according to Figure 5d–f, represented with the FFT calculation of spin ordering in the xy plane:

(4) $$FFT=\tilde{C}\left(k_{x},k_{y}\right)=\overset{N_{x}-1}{\underset{x=0}{\sum }}\overset{N_{y}-1}{\underset{y=0}{\sum }}C\left(x,y\right)e^{-i\left(\frac{2\pi xk_{x}}{N_{x}}+\frac{2\pi yk_{y}}{N_{y}}\right)}$$

where $C\left(x,y\right)=\langle S\left(0,0\right),\;S\left(x,y\right)\rangle$ is the two-spin correlation function. Since the observed in-plane spin structure consists of a down–down–down–up ordering (for $J_{7}=0.12$), there are repeating patterns over four spins in the xy plane, resulting in sharp $k_{x},\;k_{y}=\pm 0.25\;$ propagation vectors in $\tilde{C}\left(k_{x},k_{y}\right)$ (Figure 5d).

As out-of-plane interactions increase, these peaks phase into two and consecutively four peaklets. Even though the $\left|M\right|=0.5$ plateau is not observed in single-crystal HoB_4_, and our data do not exactly solve its Hamiltonian, the qualitative analysis of in-plane ordering confirms that in-plane features observed in frustrated Shastry–Sutherland magnets can indeed stem out of the out-of-plane interaction terms of the extended SSL Hamiltonian.

We notice that the treatment can produce both in-plane and out-of-plane orders but fails to reproduce the $L_{inc}=0.43$ or the one-third phase. The exact replication of in-plane and out-of-plane phases requires a thorough search of $J_{i}$ parameter space. Considering that our evaluations of the values of $J_{i}\;$ parameters were based solely on dipole–dipole interaction, the actual interaction values for the HoB_4_ single crystal can be different enough to cause the emergence of different orderings. Yet, we can attract attention to the in-plane splitting of peaks due to increasing out-of-plane couplings, shown in Figure 5d–f. These results provide a compelling idea of a scenario that in-plane orderings in an SSL magnet can be triggered via out-of-plane interactions.

We further explore the appearances of magnetization plateaus as the result of spin lattice defects, such as missing spin sites or a slight $\delta J_{i}$ shift between certain lattice points. By removing certain spins off the lattice (equivalent to creating S=0 defects arising from vacancies [44]), we study magnetization as a function of magnetic field and check the appearance of additional magnetization plateaus, which are not observed in the absence of defects. Furthermore, we consider two different sizes of defects by removing (1) individual spins from arbitrary (x, y, z) locations (we remove 1000 spins overall)—we call these single-site defects—and (2) 3×3×3-size domains from arbitrary $\left(x\pm 1,\;y\pm 1,\;z\pm 1\right)$ locations (we remove 30 domains overall)—we call these ‘chunk’ defects.

Magnetization evolution as a function of the applied field for $J_{7}=0.12$ depicts the appearance of several new magnetization plateaus in the narrow range of magnetic fields (Figure 5g). For multiple field values such as h=2.5 and h=7, it is apparent from Figure 5g (inset) that new magnetization plateaus appear when the defects are introduced. These new plateaus appear at the critical regime between $\left|M\right|=$ 0 and 0.5 at the locations $\left|M\right|\approx$ 0.05, 0.15 and 0.25. Furthermore, introducing the defects also shifts the range of the field values over which a plateau is stabilized, seen most prominently for the $\left|M\right|=0.5$ and 0.65 magnetization plateaus, which now appear at smaller fields. Defects break frustration. Besides defects having an influence on the formation of the plateaus, larger defects can start to wash out existing plateaus towards a smoother spin-glass like rise, as shown for the $\left|M\right|\approx 0.65\;$ magnetization plateau with the so-called ‘chunk’ defects. This proves that defects and their sizes play a major role, especially when comparing data from different samples and in the deduction of the truly universal features of the data.

Overall, we have shown that the simulated annealing approach of a 3D SSL lattice with defects yields qualitative explanations for the origin of some of the features of the in-plane ordering in frustrated SSL magnets. In-plane structure analysis shows that we can manipulate in-plane magnetic ordering by changing out-of-plane interactions, splitting sharp peaks into pairs and quadruplets of smaller peaklets, much as in the neutron scattering data analysis in Figure 4. Additionally, we have shown that small magnetization plateaus can appear in the presence of defects and hence can vary from sample to sample.

We emphasize that the simulated Hamiltonian assumes idealized values for interaction parameters, estimated from dipolar interactions based on interionic distances extracted from XRD data. These do not account for more complex RKKY or crystal field effects, and the results are therefore qualitative in nature. Furthermore, our calculations show that the presence of even a small concentration of defects can lead to the appearance of certain magnetization plateaus, highlighting the strong sensitivity of the formation of plateaus to sample quality. Seemingly, a larger concentration of defects leads to newer magnetization plateaus, without necessarily suppressing the original ones—hence, a sample with more plateaus could indicate a crystal with more defects. This suggests that while qualitative features such as in-plane peak splitting and plateau stabilization are robust, the exact sequence of field-induced phases may be a fingerprint of the specific defects present in the sample. Therefore, the repeatability of specific plateau features may depend on sample-specific disorder, and future work should explore this across multiple crystals with varying defect concentrations and types to isolate universal behavior from defect-driven effects.

## 6. Conclusions

This study investigated the magnetic phases and transitions in HoB_4_ using neutron diffraction, susceptibility measurements and computational modeling. A critical regime confirmed by susceptibility measurements was found to harbor a new phase with in-plane Bragg peaks in neutron diffraction. A careful analysis of this phase pointed to an in-plane peak splitting with a corresponding propagation vector ${\vec{Q}}_{\gamma }$ corresponding to a (likely commensurate) 1/7th. This result showcases the potential to discover new magnetic phases in the critical regions of phase diagrams of SSL-inspired quantum magnets and perhaps, more generally, in geometrically frustrated magnets.

The origins of in-plane ordering have been partially explained with the means of implementing the system as a 3D SSL lattice and recovering the phases with the means of annealing algorithms. As has been shown in Figure 2c, $\frac{M}{M_{sat}}\approx \frac{5}{9}$ appears at T=2 K on the magnetic phase diagram. It is interesting to note that a phase with a five-ninths magnetization was also extracted in [38] in the extended 2D Ising SSL model using a Fujitsu parallel tempering machine (Kawasaki, Japan). It remains to be seen in future work whether these phases could be related.

Our analysis of simulated annealing using a Neal simulated annealer (offered by D-Wave) indicates multiple closely spaced wave vectors arising from out-of-plane interactions that could be explained within the Ising SSL model extended to 3D. Interaction parameters $J_{i}$ ($i\epsilon \left[2,\;8\right]$; $J_{1}=1$) have been evaluated based on the interionic distances of Ho^3+^ in single-crystal HoB_4_ that were obtained with the single-crystal XRD data of our sample. The results of the simulated annealing look qualitatively similar to the peak splitting we observe in HoB_4_, although more work is required to find either the one-seventh in-plane split peaks or the one-third out-of-plane peaks observed in our data.

Furthermore, via the analysis of a defective lattice we suggest a mechanism for the formation of new plateaus and features, which have been reported by certain groups earlier close to criticality. It has been established that in-plane magnetic ordering can be affected by out-of-plane interactions and most importantly by the existence of defects that break the frustration. Variation in out-of-plane interactions splits in-plane ordering, producing (qualitatively) similar features as depicted in Figure 4. Introducing the defects in the system shows the appearance of narrow plateaus, otherwise non-existent in the SSL system. This can perhaps explain why in certain samples (possibly with some defects) additional magnetization plateaus can be observed. We have shown that defects can introduce new magnetization plateaus, especially at the critical points. In our sample, we did not notice many of the small plateaus observed in some previous reports above the one-third plateau, which might therefore suggest a sample with lesser defects. This leads to a need for caution when analyzing data from ferrimagnetic samples. Yet, the smaller magnetization plateaus could also provide a unique fingerprint for the internal structure of the crystal, which could be extracted using further sophisticated tools and AI-driven techniques.

A quantum phase transition observed in HoB_4_, with interactions beyond just the longitudinal-field Ising model, requires a complete and quantum treatment of the phase transition, which requires DMRG techniques that are beyond the purview of this manuscript. A transverse-field Ising model (TFIM) can be emulated in a Rydberg atom-based platform such as QuEra [45] or using fast-quench techniques using D-Wave quantum annealers [46] or using the Google Sycamore platform [47]. Unfortunately, any Ising quantum annealer today is incapable of embedding any out-of-plane terms in the Hamiltonian, which are required to enumerate the out-of-plane orders observed in HoB_4_. It turns out that a complete treatment of the full Hamiltonian may require some of these computing methods to come of maturity in the future. Yet, the data offers a compelling case for machine learning-based Hamiltonian discovery, where AI kernels could be trained using not only classical (ref. [48]) but also quantum treatments of the Hamiltonians, both with and without defects, and then tested on our dataset here.

## Figures and Tables

**Table 1:** Single-crystal X-ray diffraction data of HoB_4_.

T (K)		296				
Crystal system		Tetragonal				
Space group, Hall Group		*P*_4_/*mbm*, −*P* 4_2_*ab*				
a (Å), b (Å), c (Å)		7.01, 7.01, 4.01				
*α*, *β*, *γ*		90°, 90°, 90°				
Volume (Å^3^)		202.16				
Atom	Wyckoff	x	y	z	*U_iso_*(Å^2^)	occupancy
Ho	4g	0.18197	0.68197	0.00000	0.006	1.00
B	8j	0.32340	0.46120	0.50000	0.007	1.00
B	4e	0.50000	0.50000	0.20220	0.006	1.00
B	4h	0.08700	0.41300	0.50000	0.008	1.00

**Table 2:** Initial guess of interaction parameters according to dipole–dipole interactions. $r_{i}$ represents the distances between Holmium ions connected through $\;J_{i}$ interaction in a $HoB_{4}$ single crystal as measured using XRD in Section 2.

*r*_2_/*r*_1_	0.99	*J*_2_/*J*_1_	1.03
*r*_3_/*r*_1_	1.73	*J*_3_/*J*_1_	0.19
*r*_4_/*r*_1_	1.41	*J*_4_/*J*_1_	0.35
*r*_5_/*r*_1_	1.93	*J*_5_/*J*_1_	0.14
*r*_6_/*r*_1_	1.09	*J*_6_/*J*_1_	0.77
*r*_7_/*r*_1_	1.47	*J*_7_/*J*_1_	0.314
*r*_8_/*r*_1_	1.48	*J*_8_/*J*_1_	0.308

## Data Availability

The original contributions presented in the study are included in the article/Supplementary Materials, further inquiries can be directed to the corresponding authors.

## Supplementary Materials

The following supporting information can be downloaded at: https://www.mdpi.com/article/10.3390/ma18112504/s1. Figure S1: In-plane Shastry—Sutherland structure of single crystal HoB_4_; Figure S2: Magnetic susceptibility evaluation as the function of temperature for applied B = 0.05 T magnetic field; Figure S3: Normalized magnetization ($\frac{M}{M_{sat}}$) as the function of magnetic field for T = 2 K.

## References

[1] Balents L. (2010). Spin liquids in frustrated magnets. Nature.

[2] Moessner R., Ramirez A.P. (2006). Geometrical frustration. Phys. Today.

[3] Rau J.G., Lee E.K.-H., Kee H.-Y. (2016). Spin-Orbit Physics Giving Rise to Novel Phases in Correlated Systems: Iridates and Related Materials. Annu. Rev. Condens. Matter Phys..

[4] Shastry B.S., Sutherland B. (1981). Exact ground state of a quantum mechanical antiferromagnet. Phys. B+C.

[5] Miyahara S., Ueda K.J. (2003). Theory of the orthogonal dimer Heisenberg spin model for SrCu_2_ (BO_3_)_2_. Phys. Condens. Matter.

[6] Hu H.P., Cheng C., Luo H.-G., Chen S. (2015). Topological incommensurate magnetization plateaus in quasi-periodic quantum spin chains. Sci. Rep..

[7] Lacroix C., Mendels P., Mila F. (2011). Introduction to Frustrated Magnetism.

[8] Kitaev A. (2006). Anyons in an exactly solved model and beyond. Ann. Phys..

[9] Savary L., Balents L. (2017). Quantum Spin Liquids: A Review. Rep. Prog. Phys..

[10] Jensen J., Mackintosh A.R. (1991). Rare Earth Magnetism: Structures and Excitations.

[11] Dublenych Y.I. (2013). Ground States of an Ising Model on an Extended Shastry-Sutherland Lattice and the1/2-Magnetization Plateau in Some Rare-Earth-Metal Tetraborides. Phys. Rev. E.

[12] Huo L., Huang W.C., Yan Z.B., Jia X.T., Gao X.S., Qin M.H., Liu J.-M. (2013). The Competing Spin Orders and Fractional Magnetization Plateaus of the Classical Heisenberg Model on Shastry-Sutherland Lattice: Consequence of Long-Range Interactions. J. Appl. Phys..

[13] Brunt D., Balakrishnan G., Mayoh D.A., Lees M.R., Gorbunov D., Qureshi N., Petrenko O.A. (2018). Magnetisation Process in the Rare Earth Tetraborides, NdB4 and HoB4. Sci. Rep..

[14] Siemensmeyer S., Wulf E., Mikeska H.-J., Flachbart K., Gabáni S., Mat’aš S., Priputen P., Efdokimova A., Shitsevalova N. (2008). Fractional Magnetization Plateaus and Magnetic Order in the Shastry-Sutherland MagnetTmB4. Phys. Rev. Lett..

[15] Marshall M., Billingsley B.R., Bai X., Ma Q., Kong T., Cao H. (2023). Field-Induced Partial Disorder in a Shastry-Sutherland Lattice. Nat. Com..

[16] Qureshi N., Bourdarot F., Ressouche E., Knafo W., Iga F., Michimura S., Regnault L.-P., Duc F. (2022). Possible Stripe Phases in the Multiple Magnetization Plateaus in TbB4 from Single-Crystal Neutron Diffraction under Pulsed High Magnetic Fields. Phys. Rev. B.

[17] Sachdev S. (1999). Quantum Phase Transitions.

[18] Löhneysen H.v., Rosch A., Vojta M., Wölfle P. (2007). Fermi-Liquid Instabilities at Magnetic Quantum Phase Transitions. Rev. Mod. Phys..

[19] Sandvik A.W. (2010). Ground States of a Frustrated Quantum Spin Chain with Long-Range Interactions. Phys. Rev. Lett..

[20] Heidrich-Meisner F., Sergienko I.A., Feiguin A.E., Dagotto E.R. (2007). Universal Emergence of the One-Third Plateau in the Magnetization Process of Frustrated Quantum Spin Chains. Phys. Rev. B.

[21] Samarakoon A.M., Samarakoon A.M., Laurell P., Balz C., Banerjee A., Lampen-Kelley P., Mandrus D., Nagler S.E., Okamoto S., Tennant D.A. (2022). Extraction of Interaction Parameters for α−RuCl3 from Neutron Data Using Machine Learning. Phys. Rev. R.

[22] Samarakoon A.M., Alan T.D. (2020). Machine Learning for Magnetic Phase Diagrams and Inverse Scattering Problems. arXiv.

[23] Brassington A., Ma Q., Sala G., Kolesnikov A.I., Taddei K.M., Wu Y., Choi E.S., Wang H., Xie W., Ma J. (2024). Magnetic Properties of the Quasi-XY Shastry-Sutherland Magnet Er_2_Be_2_SiO_7_. Phys. Rev. Mat..

[24] Yang J.-W., Luo W.-W., Zhu W., Wang L., Yang B., Sengupta P. (2024). Chiral Spin Liquid on a Shastry-Sutherland Heisenberg Antiferromagnet. Phys. Rev. B.

[25] Corboz P., Zhang Y., Ponsioen B., Mila F. (2025). Quantum spin liquid phase in the Shastry-Sutherland model revealed by high-precision infinite projected entangled-pair states. arXiv.

[26] Zhang X.-T., Gao Y.H., Chen G. (2024). Thermal Hall Effects in Quantum Magnets. Phys. Rep..

[27] Cui Y., Du K., Wu Z., Li S., Yang P., Chen Y., Xu X., Chen H., Li C., Liu J. (2024). Two plaquette-singlet phases in the Shastry-Sutherland compound SrCu_2_(BO_3_)_2_. arXiv.

[28] Onizuka K., Kageyama H., Narumi Y., Kindo K., Ueda Y., Goto T. (2000). 1/3 Magnetization Plateau in SrCu**_2_**(BO**_3_**)**_2_**- Stripe Order of Excited Triplets -. J. Phys. Soc. Jpn..

[29] Shi Z., Dissanayake S., Corboz P., Steinhardt W., Graf D., Silevitch D.M., Dabkowska H.A., Rosenbaum T.F., Mila F., Haravifard S. (2022). Discovery of Quantum Phases in the Shastry-Sutherland Compound SrCu_2_(BO_3_)_2_ under Extreme Conditions of Field and Pressure. Nat. Com..

[30] Matsuda Y., Abe N., Takeyama S., Kageyama H., Corboz P., Honecker A., Manmana S.R., Foltin G.R., Schmidt K.P., Mila F. (2013). Magnetization of SrCu_2_(BO_3_)_2_ in Ultrahigh Magnetic Fields up to 118 T. Phys. Rev. Lett..

[31] Brunt D., Balakrishnan G., Wildes A.R., Ouladdiaf B., Qureshi N., Petrenko O.A. (2017). Field-Induced Magnetic States in Holmium Tetraboride. Phys. Rev. B.

[32] Kim J.Y., Cho B.K., Han S.H. (2009). Anisotropic Magnetic Phase Diagrams of HoB4 Single Crystal. J. Appl. Phys..

[33] Fisk Z., Maple M.B., Johnston D.C., Woolf L.D. (1981). Multiple Phase Transitions in Rare Earth Tetraborides at Low Temperature. Solid State Commun..

[34] Mat’aš S., Siemensmeyer K., Wheeler E., Wulf E., Beyer R., Hermannsdörfer T., Ignatchik O., Uhlarz M., Flachbart K., Gabáni S. (2010). Magnetism of rare earth tetraborides. J. Phys. Conf. Ser..

[35] Dublenych Y.I. (2012). Ground States of the Ising Model on the Shastry-Sutherland Lattice and the Origin of the Fractional Magnetization Plateaus in Rare-Earth-Metal Tetraborides. Phys. Rev. Lett..

[36] Farkašovský P., Regeciová L. (2019). Magnetization Plateaus and Phase Diagrams of the Extended Ising Model on the Shastry-Sutherland Lattice: Effects of Long-Range Interactions. Eur. Phys. J. B.

[37] Haravifard S., Banerjee A., Lang J.C., Srajer G., Silevitch D.M., Gaulin B.D., Dabkowska H.A., Rosenbaum T.F. (2012). Continuous and Discontinuous Quantum Phase Transitions in a Model Two-Dimensional Magnet. Proc. Natl. Acad. Sci. USA.

[38] Haravifard S., Banerjee A., van Wezel J., Silevitch D.M., dos Santos A.M., Lang J.C., Kermarrec E., Srajer G., Gaulin B.D., Molaison J.J. (2014). Emergence of Long-Range Order in Sheets of Magnetic Dimers. Proc. Natl. Acad. Sci. USA.

[39] Haravifard S., Banerjee A., van Wezel J., Silevitch D.M., dos Santos A.M., Lang J.C., Kermarrec E., Srajer G., Gaulin B.D., Molaison J.J. (2015). Reply to Zayed: Interplay of Magnetism and Structure in the Shastry–Sutherland Model. Proc. Natl. Acad. Sci. USA.

[40] Kayris P., King A.D., Ozfidan I., Boothby K., Raymond J., Banerjee A., Humble T.S. (2020). Simulating the Shastry-Sutherland Ising Model Using Quantum Annealing. Phys. Rev. X Quantum..

[41] Jha A.A., Stoyanoff E.L., Khundzakishvili G., Kairys P., Ushijima-Mwesigwa H., Banerjee A. Digital Annealing Route to Complex Magnetic Phase Discovery. Proceedings of the International Conference on Rebooting Computing (ICRC).

[42] Brunt D., Hatnean M.C., Petrenko O.A., Lees M.R., Balakrishnan G. (2019). Single-Crystal Growth of Metallic Rare-Earth Tetraborides by the Floating-Zone Technique. Crystals.

[43] Feng J.J., Huo L., Huang W.C., Wang Y., Qin M.H., Liu J.-M., Ren Z. (2014). The Main 1/2 Magnetization Plateau in Shastry-Sutherland Magnets: Effect of the Long-Range Ruderman-Kittel-Kasuya-Yosida Interaction. EPL.

[44] Biswas D., Sahadev N., Adhikary G., Balakrishnan G., Maiti K. (2013). Evolution of the Electronic Structure of HoB4with Temperature. Phys. Rev. B.

[45] Manovitz T., Li S.H., Ebadi S., Samajdar R., Geim A.A., Evered S.J., Bluvstein D., Zhou H., Koyluoglu N.U., Feldmeier J. (2025). Quantum Coarsening and Collective Dynamics on a Programmable Simulator. Nature.

[46] Salloum H., Salloum A., Mazzara M., Zykov S. (2024). Quantum Annealing in Machine Learning: QBoost on D-Wave Quantum Annealer. Procedia Comput. Sci..

[47] Castelvecchi D. (2024). “A Truly Remarkable Breakthrough”: Google’s New Quantum Chip Achieves Accuracy Milestone. Nature.

[48] Beck T., Baroni A., Bennink R., Buchs G., Antonio E., Eisenbach M., Ferreira R., Meena M.G., Gottiparthi K., Groszkowski P. (2024). Integrating Quantum Computing Resources into Scientific HPC Ecosystems. Sciencedirect.

