# Supplementary material for: Criticality and Magnetic Phases of Ising Shastry–Sutherland Candidate Holmium Tetraboride
Source: Materials (Basel). 2025 May 26;18(11):2504. doi: 10.3390/ma18112504 (PMC12155676; doi:10.3390/ma18112504)
Supplement: Supplementary file 1 [file materials-18-02504-s001.zip › materials-3642831-supplementary.pdf]

Supplemental material

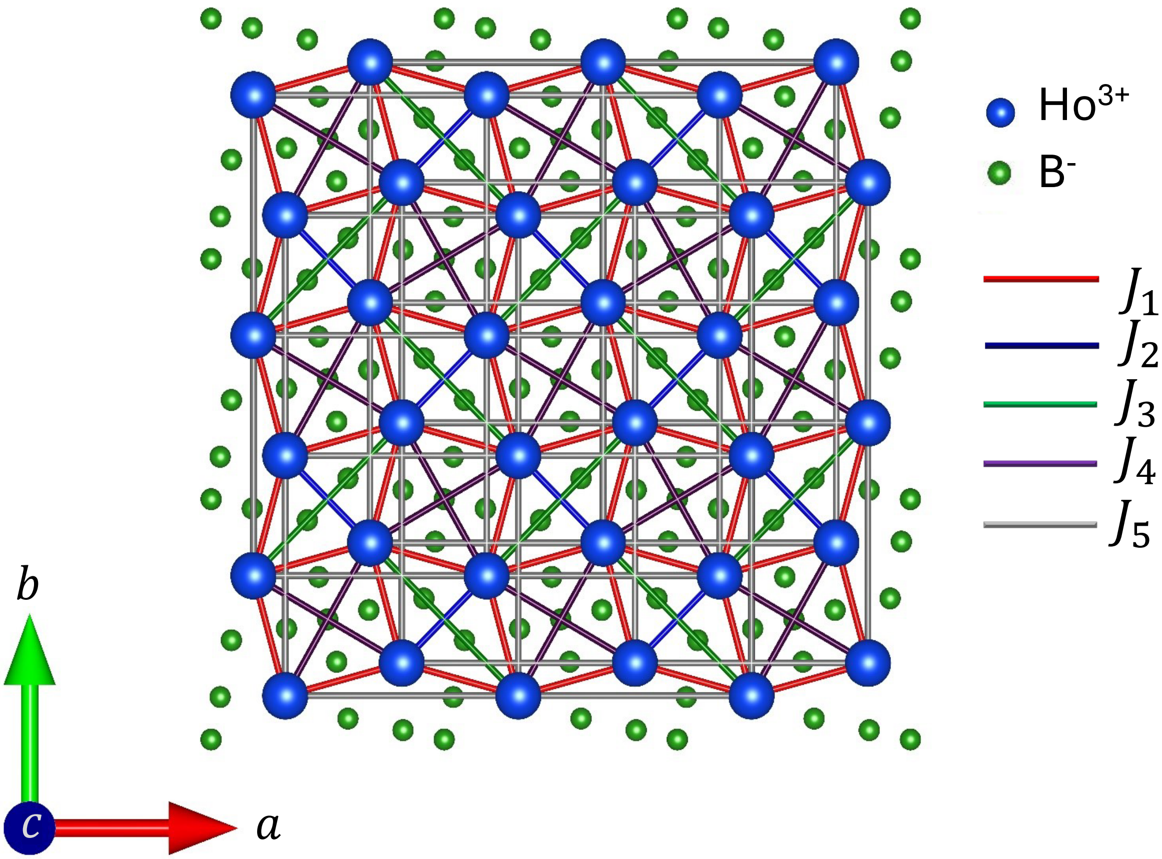

Figure S1: In-plane Shastry – Sutherland structure of single crystal HoB_4. In-plane interactions include J_1, J_2, J_3, J_4 and J_5. First two interactions represent interactions in (1), while the rest is part of extended in-plane SSL Hamiltonian discussed in [1].

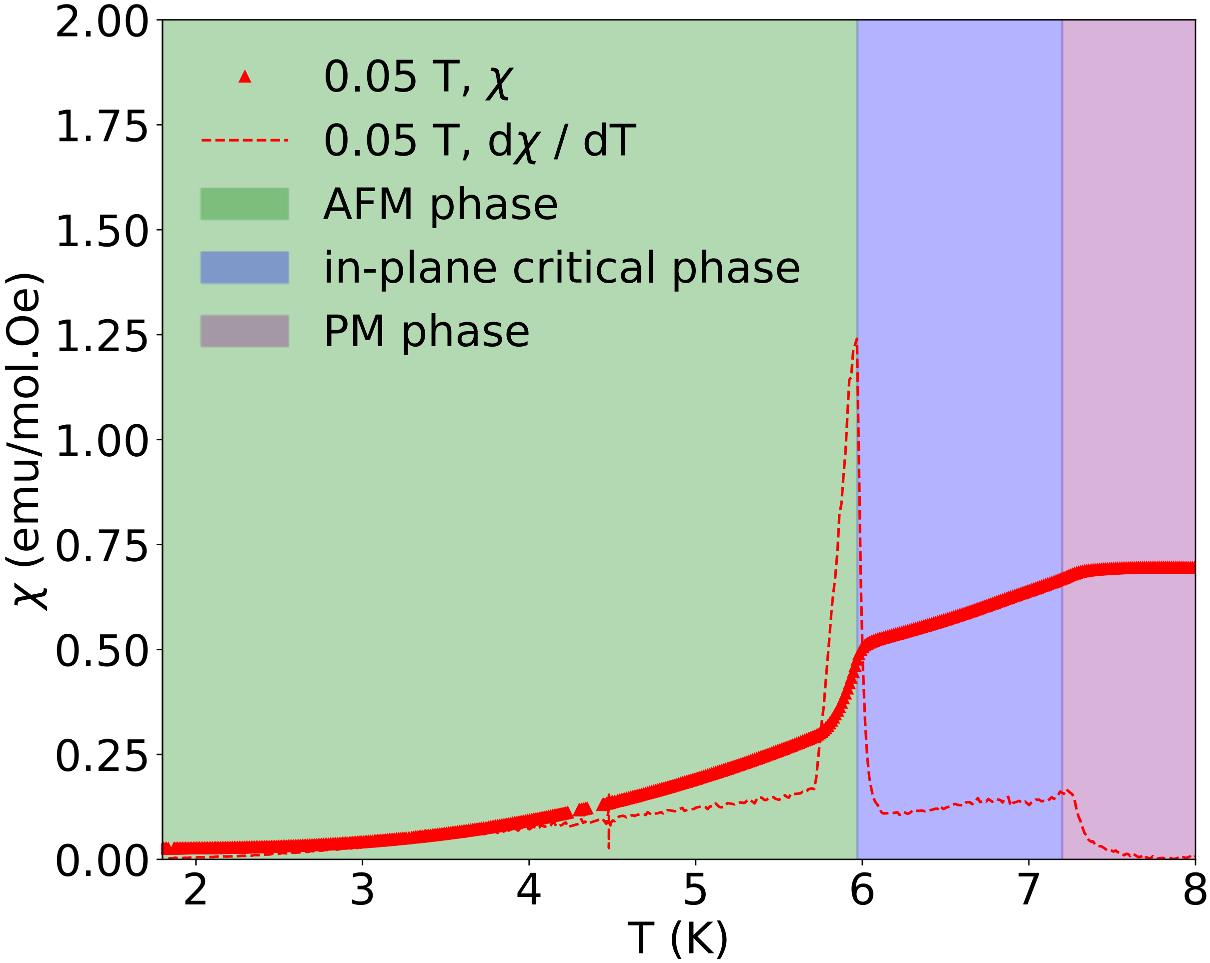

Figure S2: Magnetic susceptibility evaluation as the function of temperature for applied B = 0.05 T magnetic field. The evolution shows the transition between paramagnetic (PM), in-plane critical and antiferromagnetic (AFM) phases as the temperature goes down.

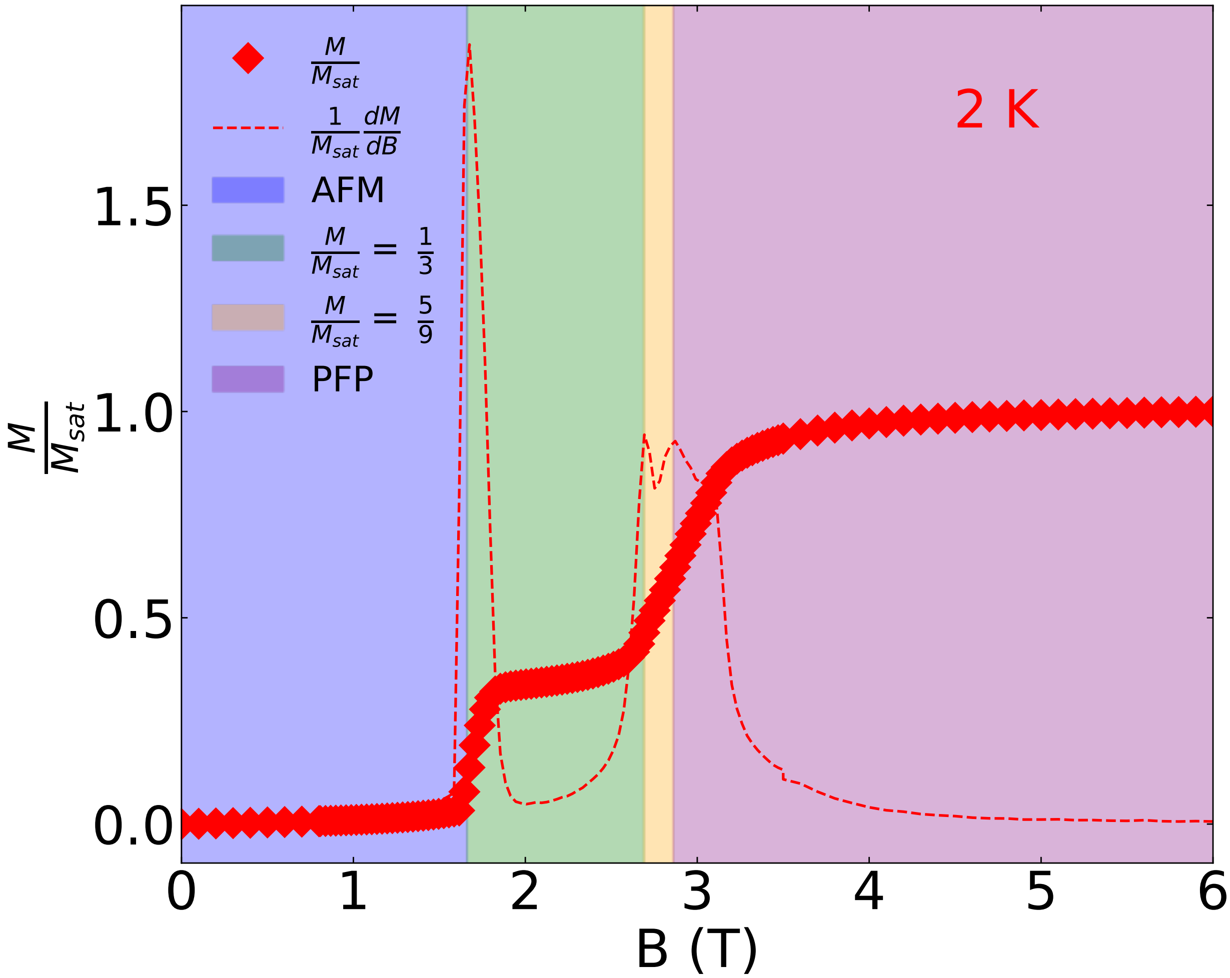

Figure S3: Normalized magnetization ($\frac{M}{M_{sat}}$) as the function of magnetic field for T = 2 K. Magnetic phase evolution consists of antiferromagnetic (AFM), out-of-plane 1/3rd ordering, trace of 5/9th ordering between magnetic field B ~ [2.7, 2.86] T and field polarized phase above 2.86 T field.

References:

1. Jha, A.A.; Stoyanoff, E. L.; Khundzakishvili, G.; Kairys, P.; Ushijima-Mwesigwa, H.; Banerjee, A. Digital Annealing Route to Complex Magnetic Phase Discovery. In Proceedings of the International Conference on Rebooting Computing (ICRC), Los Alamitos, CA, USA, 30 November–2 December 2021; pp. 119–123.
